# The association between systemic immune-inflammation index and cardiotoxicity related to 5-Fluorouracil in colorectal cancer

**DOI:** 10.1186/s12885-024-12568-0

**Published:** 2024-06-29

**Authors:** Xiaoqin Liu, Yan Wang, Wenling Wang, Hongming Dong, Gang Wang, Wanghua Chen, Juan Chen, Weiwei Chen

**Affiliations:** 1 Department of Oncology, Affiliated Hospital of Guizhou Medical University, Guiyang, China; 2 Department of Abdominal Oncology and Clinical Medicine, Affiliated Cancer Hospital of Guizhou Medical University, Guizhou Medical University, Guiyang, China

**Keywords:** Cardiotoxicity, 5-Fluorouracil, Systemic immune-inflammation index, Colorectal cancer

## Abstract

**Background and aims:**

The cardiotoxicity related to 5-Fluorouracil (5-FU) in cancer patients has garnered widespread attention. The systemic immune-inflammation index (SII) has recently been identified as a novel predictive marker for the development of cardiovascular illnesses in individuals without pre-existing health conditions. However, it remains unclear whether the levels of SII are linked to cardiotoxicity related to 5-FU. This retrospective study aims to fill this knowledge gap by examining the correlation between SII and cardiotoxicity related to 5-FU in a colorectal cancer cohort.

**Methods:**

The study comprised colorectal cancer patients who received 5-FU-based chemotherapy at the affiliated cancer hospital of Guizhou Medical University between January 1, 2018 and December 31, 2020. After adjustment for confounders and stratification by tertiles of the interactive factor, linear regression analyses, curve fitting and threshold effect analyses were conducted.

**Results:**

Of the 754 patients included final analysis, approximately 21% (*n* = 156) of them ultimately experienced cardiotoxicity related to 5-FU. Monocytes (M) was found as an influential element in the interaction between SII and cardiotoxicity related to 5-FU. In the low tertile of M (T1: M ≤ 0.38 × 10^9^/L), increasing log SII was positively correlated with cardiotoxicity related to 5-FU (Odds Ratio [OR], 8.04; 95% confidence interval [95%CI], 1.68 to 38.56). However, a curvilinear relationship between log SII and cardiotoxicity was observed in the middle tertile of M (T2: 0.38 < M ≤ 0.52 × 10^9^/L). An increase in log SII above 1.37 was shown to be associated with a decreased risk of cardiotoxicity (OR, 0.14; 95%CI, 0.02 to 0.88), indicating a threshold effect. In the high tertile of M (T3: M > 0.52 × 10^9^/L), there was a tendency towards a negative linear correlation between the log SII and cardiotoxicity was observed (OR, 0.85; 95%CI, 0.37 to 1.98).

**Conclusion:**

Our findings suggest that SII may serve as a potential biomarker for predicting cardiotoxicity related to 5-FU in colorectal cancer patients. SII is an independent risk factor for cardiotoxicity related to 5-FU with low monocytes levels (T1). Conversely, in the middle monocytes levels (T2), SII is a protective factor for cardiotoxicity related to 5-FU but with a threshold effect.

**Supplementary Information:**

The online version contains supplementary material available at 10.1186/s12885-024-12568-0.

## Introduction

Colorectal cancer is the third most common cancer and a leading cause of cancer-related mortality globally, affecting both males and females [1]. 5-Fluorouracil (5-FU) is an essential component of chemotherapy for colorectal cancer. It has a critical role in reducing the risk of disease recurrence and mortality, ultimately improving quality of life [2]. However, the adverse effects of 5-FU have attracted widespread attention, particularly cardiotoxicity, which often manifests as chest pain similar to angina, arrhythmias, electrocardiogram ST segment alterations, myocardial infarction, cardiogenic shock, and sudden cardiac death [3]. Studies have indicated that cardiotoxicity related to 5-FU may affect 1.5–21% of patients [2, 4]. Drug-induced cardiac tissue injury might result from the over activation of several inflammatory signaling pathways [5, 6]. Both oncology and cardiovascular disorders have increasingly focused on the systemic immune-inflammation index (SII). This novel biomarker, based on neutrophils, lymphocytes, and platelets, reflects local immune responses and systemic inflammation, potentially serving as an additional diagnostic and prognostic biomarker for patients with colorectal cancer in cohort studies [7–10]. Moreover, cross-sectional studies have demonstrated a noteworthy correlation between the SII and mortality, as well as cancer-related mortality, in patients with cardiovascular diseases [11–13]. Studies have shown that cardiotoxicity related to 5-FU may be predicted through early indicators such as myocardial performance index and NT-proBNP [14, 15]. However, the correlation between SII and cardiotoxicity related to 5-FU remains unclear. This retrospective cohort study aims to investigate the correlation between SII and cardiotoxicity related to 5-FU in a colorectal cancer cohort to identify a potential predictive biomarker.

## Patients and methods

### Selection of patients

This study was a retrospective cohort study. The study received approval from the institutional ethics Committee of the Affiliated Cancer Hospital of Guizhou Medical University (FZ2021-11-298), with the requirement for informed consent waived by the committee due to the use of deidentified data. The study adhered to the reporting guidelines set forth by Strengthening the Reporting of Observational Studies in Epidemiology (STROBE) [16].

**Fig. 1:**
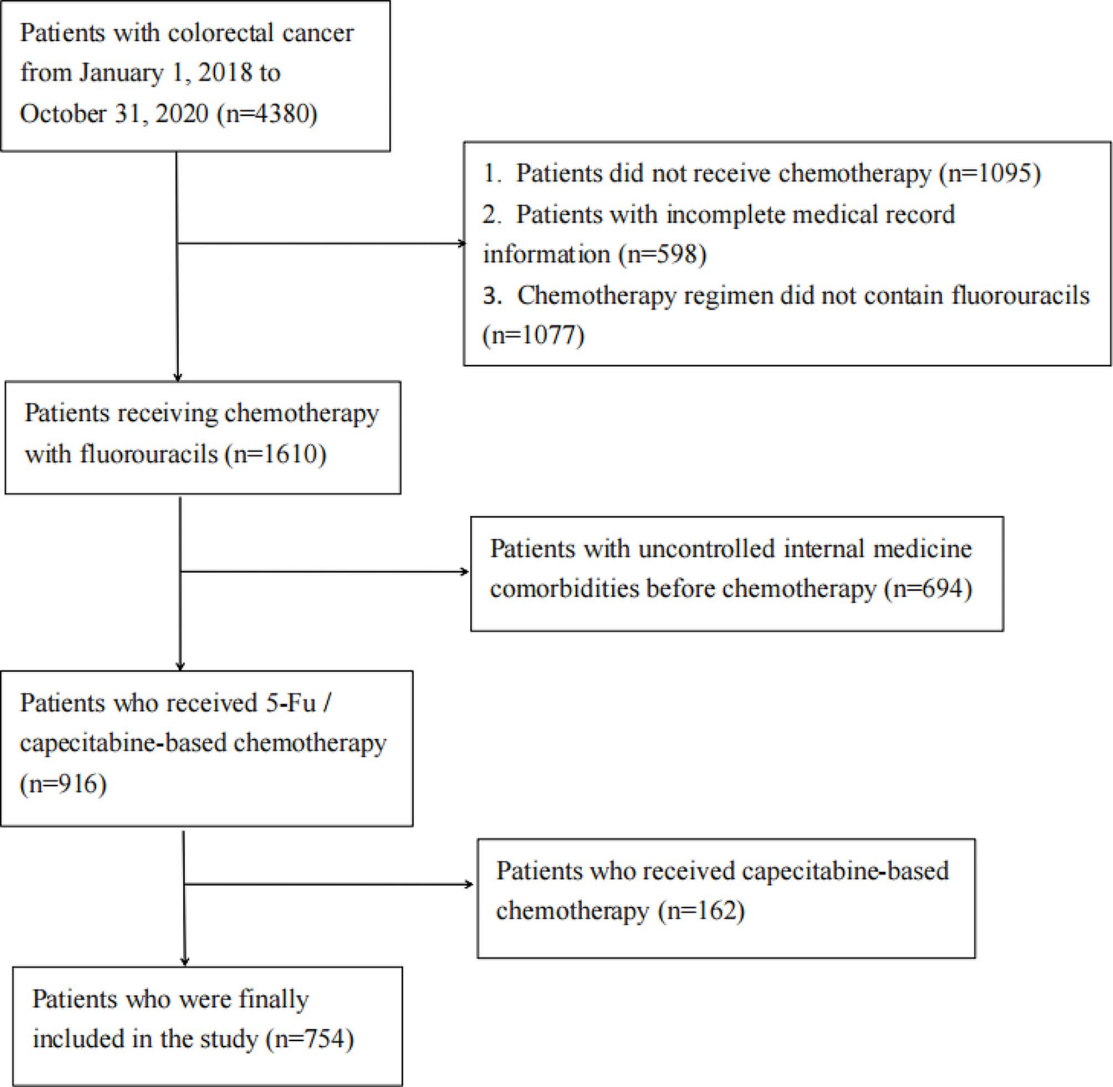
Flowchart of the participants selection

The study included patients who were diagnosed with colorectal cancer at the Affiliated Cancer Hospital of Guizhou Medical University throughout the period from January 1, 2018, to December 31, 2020. The all data was obtained through reviewing electronic medical records system. The inclusion criteria for our study were as follows: (1) Pathologically confirmed colorectal adenocarcinoma; (2) Absence of severe internal medicine comorbidities before chemotherapy (such as uncontrolled serious hypertension, serious arrhythmia, unstable angina pectoris, acute stage of myocardial infarction, or uncorrected severe cardiac insufficiency); (3) Normal blood count, liver function, renal function, and electrocardiogram, with a left ventricular ejection fraction (LVEF) ≥ 50%; (4) Patients had received a 5-FU-based chemotherapy regimen [2, 17]. Standardized interviews, physical examinations, and laboratory tests were conducted on the participants to assess their baseline characteristics. Among the initial 4380 participants, 2770 were excluded due to missing fluorouracils or incomplete medical records, 694 were excluded due to uncontrolled internal medicine comorbidities before chemotherapy, and 162 were excluded for receiving capecitabine-based chemotherapy. A total of 754 people were eventually enrolled in the study (Fig. 1).

### Variables

The data we collected for our study primarily included information on demographic factors, personal history of smoking and alcohol, internal medical comorbidities before treatment, laboratory tests within three days prior to treatment, cancer stage according to the 8th edition of the AJCC/TNM system, details of the chemotherapy regimen, chemotherapy cycles and cardiotoxicity related to 5-FU. Internal medical comorbidities included history of cardiovascular diseases (acute myocardial infarction, ischemic heart disease, hypertension, heart failure or stroke) and endocrine diseases (diabetes, hyperlipidemia, hyperthyroidism, hypothyroidism). Laboratory tests contained blood count, liver function, renal function and electrolytes.

### Exposure variable

The automated hematology analyzing devices (CoulterDxH 800 Analyzer) were used to measure the lymphocytes, neutrophils, and platelets. The SII level was determined based on prior research, which was documented as follows: SII index (×10^9^/L) = neutrophils (×10^9^/L) × platelets (×10^9^/L)/lymphocytes (×10^9^/L) [12, 18].

### Outcome variable

All patients who did not experience cardiotoxicity related to 5-FU were followed up until four weeks following completion of treatment, while patients who experienced cardiotoxicity were followed up until outcome diagnosis.

Cardiotoxicity related to 5-FU is characterized by the appearance of new symptoms that are likely to cardiac-origin symptoms, such as chest pain, palpitations, dyspnea, or cardiogenic shock. It can also be identified by further abnormal findings on electrocardiograms, such as ST-T changes or arrhythmias, as well as abnormalities on echocardiograms or an obvious increase in myocardial enzymes. These symptoms and abnormalities were discovered during the period of 5-FU treatment or within four weeks following completion of treatment [19–21]. Moreover, the possibility that the new symptoms (chest pain, palpitations, dyspnea) are caused by other bodily systems, such as the respiratory or digestive system, has been eliminated. Cardiologists conducted additional evaluations and performed required auxiliary exams to confirm the diagnosis of cardiotoxicity related to 5-FU in cases that raised suspicion.

### Covariates

The covariates used in this study are listed in detail. Variables that have been emphasized in prior research on factors linked to cardiotoxicity, as well as variables deemed significant based on our clinical knowledge, were included as potential confounders [22]. Finally, the confounding variables identified for our study included sex, age, cancer stage, history of cardiovascular diseases, history of endocrine diseases, history of smoking, use of targeted drug, dosage of 5-FU, chemotherapy cycles, alanine aminotransferase (ALT), and serum creatinine (SCR).

### Statistical analysis

Summary statistics of baseline characteristics of all patients were expressed as frequency and percentage for categorical variables, mean ± SD or median (Q1- Q3) for continuous variables. To examine variances in characteristics between the presence or absence of cardiotoxicity associated with 5-FU, we utilized the independent samples t-test (for normal distribution), Mann-Whitney U test (for non-normal distribution), or chi-square test (for categorical variables).

The base 10 log-transformed SII (log SII) conforms to a normal distribution (*P* < 0.01), and in combination with clinical significance, log SII serves as the independent variable, while the dependent variable is cardiotoxicity related to 5-FU. In our study, after conducting an interaction test, we identified a statistically significant interaction (*p* < 0.05) between monocytes and log SII on cardiotoxicity related to 5-FU. After adjustment for confounders and stratification by tertiles of the interactive factor, linear regression analyses, curve fitting and threshold effect analyses were performed. Finally, a logarithmic likelihood ratio test (LRT test) was performed to determine the presence of a threshold effect.

Statistical analyses were conducted using R software version 4.2.0 (http://www.R-project.org), with all *P* values determined through two-tailed tests of statistical significance at a 5% type I error rate.

## Results

At the last follow-up in January 2021, the study enrolled 754 patients. Of these, 156 patients (21%) developed cardiotoxicity related to 5-FU. The average time for outcome diagnosis in our study was the 3.14th cycle of 5-FU chemotherapy for the patients. Table 1 displays the baseline characteristics of the patients. Significant disparities in baseline characteristics were noted between the groups that experienced cardiotoxicity related to 5-FU and those that did not, including age, dosage of 5-FU, chemotherapy cycles, percentage of neutrophils (NE%), lymphocytes (LY), prognostic nutritional index (PNI), SCR, history of cardiovascular diseases, and use of targeted drug.

**Table 1:** ^a^Baseline characteristics of participants

Variables	Patients without cardiotoxicity (*n* = 598)	Patients with cardiotoxicity (*n* = 156)	*P*
Age (year)	54.31 ± 12.30	57.62 ± 12.62	0.003
BMI (kg/m^2^)	23.46 ± 26.70	22.83 ± 3.55	0.768
SBP (mmHg)	117.90 ± 11.90	119.73 ± 12.60	0.092
DBP (mmHg)	73.48 ± 7.48	73.43 ± 8.13	0.935
Dosage of 5-FU (g)	20.53 ± 12.70	12.32 ± 8.57	< 0.001
Chemotherapy cycles	4.96 ± 2.85	3.14 ± 2.15	< 0.001
WBC (×10^9^/L)	6.02 ± 2.12	6.26 ± 2.21	0.219
NE (×10^9^/L)	3.74 ± 1.83	4.04 ± 1.96	0.071
NE%	60.30 (52.60–67.40)	63.20 (55.20-69.73)	0.006
LY (×10^9^/L)	27.47 ± 9.89	25.28 ± 9.49	0.014
LY%	1.56 ± 0.59	1.49 ± 0.56	0.217
M (×10^9^/L)	0.49 ± 0.20	0.50 ± 0.19	0.778
EOS (×10^9^/L)	0.28 ± 0.31	0.28 ± 0.23	0.773
EOS%	6.21 ± 5.83	6.31 ± 5.58	0.848
HGB (g/L)	124.72 ± 19.91	126.49 ± 22.59	0.338
PLT (×10^9^/L)	241.25 ± 88.18	235.60 ± 77.13	0.465
NLR	0.19 ± 0.22	0.22 ± 0.24	0.087
PLR	10.76 ± 8.61	11.60 ± 8.47	0.273
SII	51.10 ± 81.20	57.59 ± 78.60	0.371
PIV	31.47 ± 68.49	33.26 ± 54.98	0.762
NAR	0.09 ± 0.05	0.10 ± 0.05	0.074
MLR	0.02 ± 0.02	0.03 ± 0.02	0.178
PNI	177.25 (144.22-208.48)	170.57 (137.04–193.50)	0.017
AST (u/L)	23.67 ± 14.83	25.49 ± 22.54	0.227
ALT (u/L)	22.68 ± 20.76	25.05 ± 39.05	0.302
ALB (g/L)	41.62 (38.80-44.14)	41.25 (38.55–43.90)	0.493
LDH (u/L)	201.52 ± 125.76	218.42 ± 166.65	0.165
GLU (mmol/L)	4.96 ± 1.12	5.10 ± 1.17	0.158
UA (µmol/L)	201.52 ± 125.76	219.18 ± 166.79	0.147
SCR (µmol/L)	66.39 ± 15.52	70.29 ± 18.27	0.007
BUN/SCR	0.07 ± 0.02	0.07 ± 0.02	0.536
K (mmol/L)	3.99 ± 0.42	3.99 ± 0.41	0.982
CA (mmol/L)	2.35 (2.26–2.45)	2.33 (2.24–2.43)	0.051
Sex			0.582
male	369 (61.71%)	100 (64.10%)	
female	229 (38.29%)	56 (35.90%)	
History of cardiovascular diseases			0.015
no	486 (81.27%)	113 (72.44%)	
yes	112 (18.73%)	43 (27.56%)	
History of endocrine diseases			0.569
no	491 (82.11%)	125 (80.13%)	
yes	107 (17.89%)	31 (19.87%)	
History of drink			0.911
no	325 (54.35%)	84 (53.85%)	
yes	273 (45.65%)	72 (46.15%)	
History of smoking			0.510
no	312 (52.17%)	86 (55.13%)	
yes	286 (47.83%)	70 (44.87%)	
Chemotherapy regimen			0.289
multidrug	575 (96.15%)	147 (94.23%)	
single drug	23 (3.85%)	9 (5.77%)	
Use of targeted drug			< 0.001
no	494 (82.61%)	101 (64.74%)	
anti-VEGFR	78 (13.04%)	44 (28.21%)	
cetuximab	26 (4.35%)	11 (7.05%)	
Cancer stage			0.230
I	13 (2.17%)	3 (1.92%)	
II	111 (18.56%)	19 (12.18%)	
III	294 (49.16%)	78 (50.00%)	
IV	180 (30.10%)	56 (35.90%)	

^a^Abbreviations: BMI, body mass index; SBP, systolic blood pressure; DBP, diastolic blood pressure; WBC, white blood cells; NE, neutrophils; NE%, percentage of neutrophils; LY, lymphocytes; LY%, percentage of lymphocytes; M, monocytes; EOS, eosinophils; EOS%, percentage of eosinophils; HGB, hemoglobin; PLT, platelets; NLR, neutrophils to lymphocytes ratio (= NE/LY); PLR, platelets to lymphocytes ratio (= PLT/LY); SII, systemic immune-inflammation index (= (NE×PLT)/LY); PIV, Pan-Immune-Inflammation value (= (NE×PLT×M) /LY); NAR, neutrophil-to-albumin ratio (= NE/ALB); MLR, monocytes to lymphocytes ratio (= M/LY); PNI, prognostic nutritional index (= ALB + 5×PLT×LY); AST, glutamic oxaloacetic transaminase; ALT, alanine aminotransferase; ALB, albumin; LDH, lactate dehydrogenase; GLU, random blood glucose; UA, uric acid; SCR, serum creatinine; BUN, urea nitrogen; K, serum potassium; CA, serum calcium

Linear regression analyses were formulated both with and without adjustments for the confounding variables. To investigate an independent association between log SII and cardiotoxicity related to 5-FU, three models were utilized. As shown in Table 2, the results were presented by monocytes tertiles (T): T1: M ≤ 0.38 × 10^9^/L; T2: 0.38 < M ≤ 0.52 × 10^9^/L; T3: M > 0.52 × 10^9^/L. In the unadjusted model, each increase in log SII was associated with a 3.92-fold increased risk of cardiotoxicity in T1 (OR, 3.92; 95%CI, 1.46 to 10.53). Meanwhile, in model I, an increase in log SII resulted in a 6.18-fold increase in the risk of cardiotoxicity in T1 (OR, 6.18, 95% CI: 1.87 to 20.46). In model II, the risk of cardiotoxicity increased by 8.04-fold (OR, 8.04; 95% CI, 1.68 to 38.56) with each additional log SII in T1. The ORs for T2 and T3 were not statistically significant in any of the three models.

**Table 2:** Risk association between log SII and cardiotoxicity related to 5-FU.

monocytes tertiles (T)	Unadjusted Mode	Model I^a^	Model II ^b^
T1 (M ≤ 0.38 × 10^9^/L)	3.92 (1.46, 10.51) 0.0066	6.18 (1.87, 20.46) 0.0028	8.04 (1.68, 38.56) 0.0092
T2 (0.38 < M ≤ 0.52 × 10^9^/L)	0.97 (0.43, 2.19) 0.9320	0.61 (0.24, 1.54) 0.2975	0.66 (0.23, 1.85) 0.4282
T3 (M > 0.52 × 10^9^/L)	1.06 (0.53, 2.12) 0.8641	0.88 (0.42, 1.81) 0.7195	0.85 (0.37, 1.98) 0.7123

Data expressed as Odds ratio (95%CI) P

^a^ Model I adjusted for sex, age, stage, history of cardiovascular diseases, history of endocrine disease, smoking, ALT, and SCR

^b^ Model II adjusted for model I plus targeted medication use, chemotherapy dosage and cycles

**Fig. 2:**
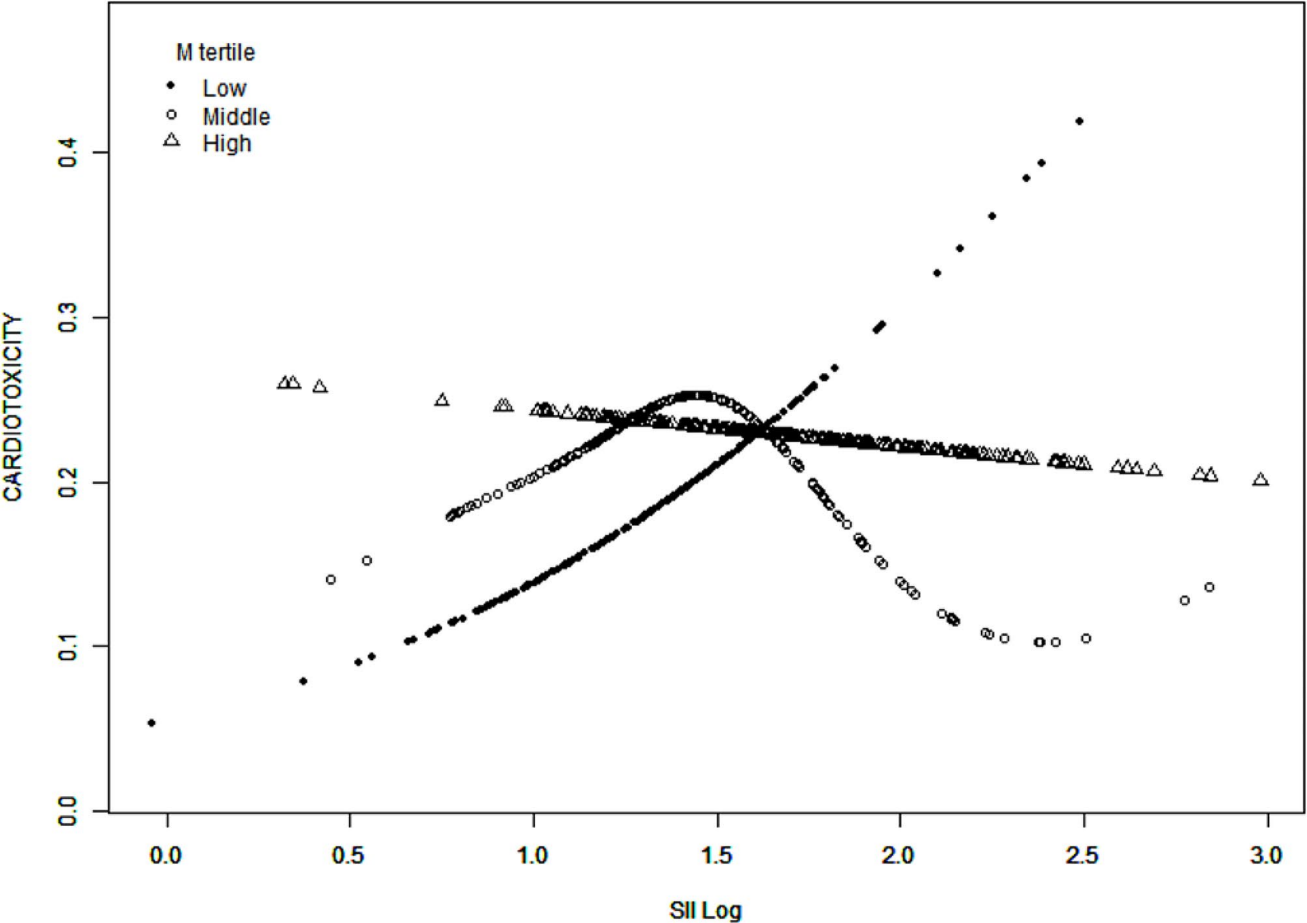
^a^Illustration of smooth curve fitting. ^a^Low M tertile = T1 (M ≤ 0.38 × 10^9^/L), Middle M tertile = T2 (0.38 < M ≤ 0.52 × 10^9^/L), High M tertile = T3 (M > 0.52 × 10^9^/L), SII Log = log SII

Smooth curve fitting and threshold effect analysis were performed after the adjustment of all confounding variables, as illustrated in Fig. 2; Table 3. In the T1, a positive linear relationship (OR, 8.04; 95%CI, 1.68 to 38.56) existed between log SII and cardiotoxicity related to 5-FU. The LRT test *P* value was 0.021 in the T2, thus a non-linear relationship and a breakpoint was exhibited. The breakpoint (K) was identified as log SII = 1.37 (SII = 23.44), which is situated between the 25th and 50th percentile of SII in the T2. When the log SII was greater than 1.37, each increase in log SII was associated with an 86% decrease in the risk of cardiotoxicity related to 5-FU, with a threshold effect (OR, 0.14; 95% CI, 0.02 to 0.88). Whereas when log SII was below 1.37, a trend of positive correlation (OR, 10.13; 95%CI, 0.68 to 150.41) is observed between log SII and cardiotoxicity related to 5-FU. In the T3, the analysis showed a negative linear correlation trend (OR, 0.85; 95%CI, 0.37 to 1.98). The same results were obtained when SII values were transformed using the natural logarithm.

**Table 3:** Threshold effect analysis after adjustment of the confounding factors

		T1 (M ≤ 0.38 × 10^9^/L)	T2 (0.38 < M ≤ 0.52 × 10^9^/L)	T3 (M > 0.52 × 10^9^/L)
Model I^a^		8.04 (1.68, 38.56) 0.0092	0.66 (0.23, 1.85) 0.4282	0.85 (0.37, 1.98) 0.7123
Model II^b^	Breakpoint (K)	1.12	1.37	2.43
	< K effect 1	0.66 (0.01, 48.74) 0.8512	10.13 (0.68, 150.41) 0.0926	1.02 (0.41, 2.54) 0.9607
	> K effect 2	18.32 (2.19, 152.91) 0.0072	0.14 (0.02, 0.88) 0.0359	0.00 (0.00, 131979.75) 0.3910
	effect 2 − 1	27.64 (0.11, 6744.54) 0.2366	0.01 (0.00, 0.65) 0.0293	0.00 (0.00, 159176.48) 0.3946
	Model fit value at K	-2.01 (-2.60, -1.42)	-1.17 (-1.64, -0.70)	-0.95 (-1.58, -0.31)
	LRT test	0.244	0.021	0.245

Data expressed as OR (95%CI) P value, and all models adjusted for sex, age, stage, history of cardiovascular diseases, history of endocrine disease, smoking, ALT, SCR, targeted medication use, chemotherapy dosage and cycles

^a^Model I was created by using a linear regression model for data fitting

^b^Model II was developed by using a piecewise regression model for data fitting

## Discussion

Inflammation is increasingly recognized as having an impact on the development and prognosis of cardiovascular diseases and cancer patients [23]. Research suggests that cancer can increase the risk of cardiovascular diseases, mostly due to inflammation, a prothrombotic state, and oxidative stress [24]. Therefore, it is imperative to examine the relationship between inflammation and cardiovascular adverse events related to chemotherapy in colorectal cancer patients, with the aim of providing more precise guidance for clinical management.

Our study has identified monocytes as an interactive factor, suggesting that the SII may be just one element in a pathway that causes damage to the heart [25]. The association between SII and cardiotoxicity related to 5-FU across different monocytes levels was observed. The stratification analysis revealed that elevating SII is an independent risk factor (OR, 8.04; 95%CI, 1.68 to 38.56) for cardiotoxicity related to 5-FU with low monocytes levels (T1). However, a protective role (OR, 0.14; 95%CI, 0.02 to 0.88) of SII was found in relation to middle monocytes levels (T2). Our results indicated that SII may aid in predicting the onset of cardiotoxicity related to 5-FU. The finding of curvilinear relationship and a breakpoint (log SII = 1.37) in the T2 is consistent with previous studies that indicate a non-linear relationship between SII and the development and prognosis of cardiovascular diseases [11, 26].

As a comprehensive indicator of systemic inflammation, the SII is commonly utilized in conjunction with monocytes to evaluate the immune status of the body comprehensively. Inflammatory tissues possess the capacity to attract monocytes from the circulatory system into injured tissues. Under the impact of several inflammatory stimuli, monocytes undergo a transformation into macrophages, which play an active role in regulating and carrying out immunological responses [27]. Studies have demonstrated that the heart contains both resident macrophages and monocyte-derived macrophages recruited to the area. Resident macrophages in the heart constitute an inherent population capable of self-maintenance and renewal, while monocyte-derived macrophages can replenish depleted tissue-resident macrophages resulting from heart injury or aging. Monocytes and macrophages play a pivotal role in cardiac injury and repair processes by acting as scavenger cells responsible for clearing cellular debris [28, 29]. Further research suggests that directing macrophage polarization effectively aids in cardiac healing in mouse models of myocardial infarction [30]. Studies targeting macrophage phagocytic receptors have revealed that low efficiency in clearing cellular debris can lead to impaired wound healing and decreased cardiac function [31, 32]. Diminished levels of monocytes indicate decreased clearance ability and relatively lower anti-inflammatory capacity within the body. Therefore, in the presence of myocardial inflammation, elevated SII levels increase the risk of cardiotoxicity related to 5-FU. However, when monocytes are relatively abundant (0.38 < M ≤ 0.52 × 10^9^/L), the body possesses a certain level of anti-inflammatory capacity. In such cases, an increase in SII levels within a certain range is a normal response of the body. This inflammatory state has not reached a harmful level for the tissues, and a certain number of monocytes can enhance the phagocytosis of cellular debris through the interaction of inflammatory factors and other immune cells, thus promoting the repair of cell damage [28]. This may explain the curvilinear relationship in T2.

Our study found that the role of SII in cardiotoxicity related to 5-FU is related to the stratification of monocytes. Based on this study, we can explore the role of monocytes / inflammatory status in cardiotoxicity related to 5-FU. Furthermore, in the clinical practice of using 5-FU, it is necessary to closely monitor the inflammatory status of patients in order to enable early prevention, monitoring, and management of cardiotoxicity related to 5-FU, especially when monocyte levels are low (T1:M ≤ 0.38 × 10^9^/L). A recent study has confirmed the close association between SII levels and the development of cardiovascular diseases, including hypertension, heart failure, and acute myocardial infarction [33–35]. In a meta-analysis conducted by Ye et al. in 2022, which encompassed 152,996 patients across 13 studies, it was observed that elevated SII levels were linked to an increased risk of cardiovascular diseases such as ischemic stroke, hemorrhagic stroke, and myocardial infarction [36]. Additionally, SII has the potential to predict the severity of coronary artery disease and acute ischemic stroke [37]. Therefore, SII not only serves as a significant clinical marker for cardiotoxicity related to 5-FU in colorectal cancer, but it may also be associated with adverse cardiovascular events in the general population.

Our study delved into and examined non-linearity and the threshold effect by stratifying the interactive factor into tertiles. This study is a retrospective cohort study, which inherently has limitations. However, we have taken measures to address these limitations by adjusting relevant variables to minimize any potential confounding effects. Despite these efforts, there are still some limitations to consider. Firstly, the patients for this study were selected from a single center, which may limit the generalizability of our conclusions to other hospitals or regions. Multiple similar studies in future trials will be required to validate our findings. Secondly, our study only examined baseline SII levels, and it would be beneficial for future studies to investigate changes in SII during the follow-up period. Third, capecitabine, a predrug form of 5-FU, is considered to have better safety, tolerability and intratumor drug concentration levels [38]. Thus, it would be valuable to explore whether the outcomes of our study can be applied to colorectal cancer patients undergoing capecitabine-based chemotherapy in future trials.

## Conclusion

In conclusion, our study demonstrated that the association between SII and cardiotoxicity related to 5-FU in colorectal cancer has U-shape relationship and different impacts at various monocytes levels. SII is an independent risk factor for cardiotoxicity related to 5-FU with low monocytes levels (T1: M ≤ 0.38 × 10^9^/L). Conversely, in the middle monocytes levels (T2: 0.38 < M ≤ 0.52 × 10^9^/L), SII serves as a protective factor for cardiotoxicity related to 5-FU but with threshold effect. Our results raise the possibility that SII may be used as a biomarker for predicting the development of cardiotoxicity related to 5-FU in colorectal cancer patients.

### Electronic supplementary material

Below is the link to the electronic supplementary material.

Supplementary Material 1

## Data Availability

The datasets supporting the conclusions of this article are included within the article and its additional file. Further inquiries can be directed to the corresponding author.

## Abbreviations

5-FU: 5-fluorouracil
SII: systemic immune-inflammation index
M: Monocytes
OR: Odds Ratio
95%CI: 95% confidence interval
log SII: log-transformed SII
LRT test: logarithmic likelihood ratio test
BMI: body mass index
SBP: systolic blood pressure
DBP: diastolic blood pressure
WBC: white blood cells
NE: neutrophils
NE%: percentage of neutrophils
LY: lymphocytes
LY%: percentage of lymphocytes
EOS: eosinophils
EOS%: percentage of eosinophils
HGB: hemoglobin
PLT: platelets
NLR: neutrophils to lymphocytes ratio (= NE/LY)
PLR: platelets to lymphocytes ratio (= PLT/LY)
SII: systemic immune-inflammation index (= (NE×PLT)/LY)
PIV: Pan-Immune-Inflammation value (= (NE×PLT×M) /LY)
NAR: neutrophil-to-albumin ratio (= NE/ALB)
MLR: monocytes to lymphocytes ratio (= M/LY)
PNI: prognostic nutritional index (= ALB + 5×PLT×LY)
AST: glutamic oxaloacetic transaminase
ALT: alanine aminotransferase
ALB: albumin
LDH: lactate dehydrogenase
GLU: random blood glucose
UA: uric acid
SCR: serum creatinine
BUN: urea nitrogen
K: serum potassium
CA: serum calcium
K: breakpoint
